# Novel Biomarkers of Heart Failure in Pediatrics

**DOI:** 10.3390/children9050740

**Published:** 2022-05-18

**Authors:** Teja Senekovič Kojc, Nataša Marčun Varda

**Affiliations:** 1 Department of Perinatology, University Medical Centre Maribor, Ljubljanska 5, 2000 Maribor, Slovenia; 2 Department of Paediatrics, University Medical Centre Maribor, Ljubljanska 5, 2000 Maribor, Slovenia; natasa.marcunvarda@siol.net; 3 Medical Faculty, University of Maribor, Taborska 8, 2000 Maribor, Slovenia

**Keywords:** biomarkers, heart failure, myocardial stretch, myocyte injury, myocardial remodeling, inflammation, renal dysfunction, oxidative stress, child

## Abstract

Novel biomarkers of heart failure are the subject of numerous studies. Biomarkers of heart failure can be determined in the blood and in the urine. Seven groups of biomarkers of heart failure based on pathophysiological mechanisms are presented in this review, namely biomarkers of myocardial stretch, myocyte injury, myocardial remodeling, biomarkers of inflammation, renal dysfunction, neurohumoral activation, and oxidative stress. Studies of biomarkers in the pediatric population are scarce, therefore, further investigation is needed for reliable prognostic and therapeutic implications. The future of biomarker use is in multimarker panels that include a combination of biomarkers with different pathophysiological mechanisms in order to improve their diagnostic and prognostic predictive value.

## 1. Introduction

Despite advances in medicine, heart failure is still an important cause of morbidity and mortality in the modern world. Consequently, there is a considerable need to find new ways of predicting, screening, and prognosticating heart failure, especially in pediatrics [1]. Laboratory diagnostics is an important part of the decision-making process in everyday clinical practice in order to come to a diagnosis, and additionally for risk stratification and therapeutical choices [2].

Various novel biomarkers of heart failure have been studied in adults. However, reliable novel biomarkers of heart failure in pediatrics have not been sufficiently studied for everyday clinical practice yet, therefore, additional knowledge is very welcome. In this review, we try to classify biomarkers according to the pathophysiological mechanisms that contribute to the development of heart failure. Several biomarkers of heart failure are still under evaluation and a detailed review of all of them is beyond the scope of this narrative review.

In pediatrics, biomarkers of heart failure are particularly important for the early identification and risk stratification of patients with systemic diseases and associated risk for early development of heart failure. Good biomarkers have the following characteristics: high sensitivity and specificity, the possibility of simultaneous processing of many samples, short analysis time, low cost, and good clinical applications, thus predicting the risk of heart failure and the associated prognosis as well as the adequacy of monitoring [3].

Two strategies are currently in place to detect newer biomarkers of heart failure, the first is based on proteomics and metabolomics, which means comparing blood and tissue samples from patients with heart failure with healthy individuals. It provides data on the expression of proteins and their breakdown products [4]. This first approach does not provide a lot of information about the pathophysiological processes that lead to the disease, which is typical for the second approach, based on the mechanisms underlying the development of cardiovascular disease [5]. Biomarkers of heart failure can be determined in blood samples and some also in urine samples. In this review, we will present seven groups of newer biomarkers that are associated with heart failure based on pathophysiological mechanisms, as seen in Table 1. Normal values of some biomarkers of heart failure are presented in Table 2 [6,7,8,9]. In addition, we will also highlight the possibilities of determining biomarkers in the urine, which allows less invasive sampling and better participation of patients and healthy individuals in potential clinical studies.

## 2. Biomarkers of Myocardial Stretch

**Figure 1:**
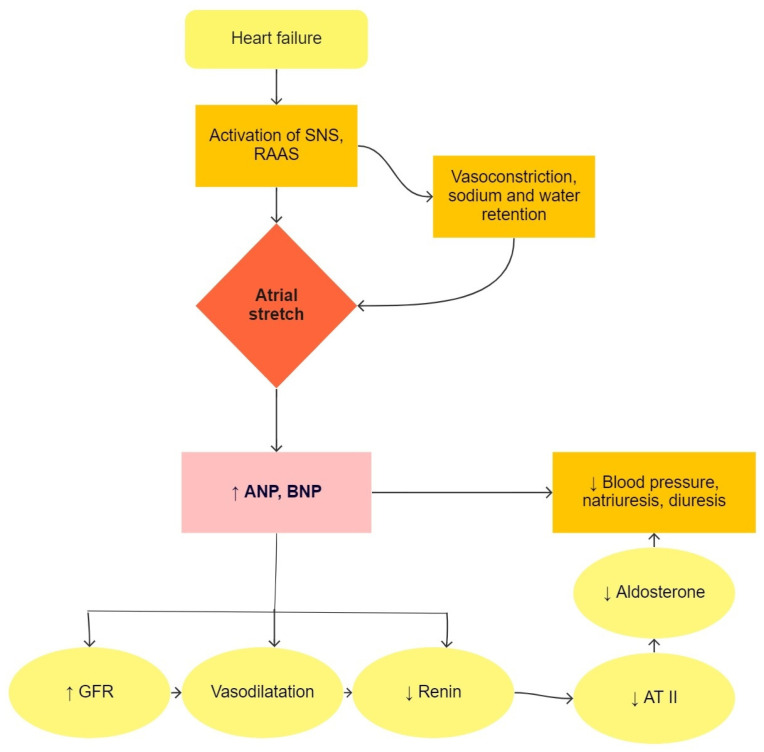
The physiological function of natriuretic peptides in heart failure. SNS, sympathetic nervous system; RAAS, renin-angiotensin-aldosterone system; ANP, atrial natriuretic peptide; BNP, brain natriuretic peptide; GFR, glomerular filtration rate; AT II, angiotensin II.

Heart failure is a condition in which the heart is not able to pump enough blood to meet the needs of all tissues [5]. This causes an increase in blood volume by regulating sodium and retaining water in the body. Natriuretic peptides are produced in atrial and ventricular cells due to pressure or volume overload, as seen in Figure 1. ANP (atrial natriuretic peptide) and BNP (brain natriuretic peptide) are used in the diagnosis of heart failure and lead to natriuresis, diuresis, and vasodilatory mechanisms, which are compensatory mechanisms in heart failure [10]. In clinical practice, the precursor of BNP, i.e., NT-proBNP (N-terminal-proBNP), is used primarily in suspected heart failure and in the monitoring of patients with known heart failure. BNP and NT-proBNP values are influenced by age, sex, obesity, renal function, and lung disease [11]. In pediatric patients, NT-proBNP correlates well with the stage of disease and is a better predictive factor of heart failure than BNP [12].

### Midregional Proatrial Natriuretic Peptide (MR-proANP)

Mid-regional proatrial natriuretic peptide (MR-proANP) is an atrial natriuretic peptide that is determined in the form of a prohormone because of its longer half-life and greater plasma stability. Recent research has identified the middle region of prohormone or MR-proANP [13]. N-terminal pro-B-type natriuretic peptide (NT-proBNP) and MR-proANP have a good correlation with left ventricular ejection fraction [14]. The combined use of both biomarkers improves the chances of diagnosing and predicting heart failure [15]. Compared to BNP, MRproANP has a better diagnostic and prognostic value for heart failure in obese patients and patients with renal dysfunction. The lack of reliable data and laboratory costs are the main limitations for clinical use of MR-proANP compared to other natriuretic peptides [16,17].

## 3. Biomarkers of Myocyte Injury

Numerous studies have already been conducted in the field of biomarkers of myocardial damage, as the process of cell death of cardiomyocytes due to apoptosis or necrosis is at the forefront of heart failure. Various mechanisms lead to cell death, such as poorer tissue perfusion, poorer oxygen supply, increased heart muscle load, circulating neurohormones, adrenergic system activation, inflammation, and oxidative stress [18].

### 3.1. High-Sensitivity Cardiac Troponin (hs-cTn)

Troponins are regulatory proteins involved in the contractions of the heart muscle and skeletal muscle. Cardiac troponins are organized as a troponin complex consisting of different subunits. Troponin C binds calcium, troponin I inhibits contraction, and troponin T promotes contraction through the binding of the troponin complex to tropomyosin [19]. Cardiac troponin C is present in cardiac muscle and skeletal muscle, while cardiac troponin I and cardiac troponin T are specific to cardiac muscle. With myocardial injury, cardiac troponin T is released slowly within a few days to two weeks after injury, whereas cardiac troponin I is released rapidly, most often within two hours after injury [20]. Cardiac troponins are most useful in the detection of patients with myocardial ischemia, for example in acute coronary syndrome [16]. At the same time, studies have shown that cardiac troponin I occurs in the plasma of patients with heart failure without myocardial ischemia [21]. Cardiac troponin I correlates well with left ventricular ejection fraction and with mortality in patients with heart failure [22]. On the other hand, elevated cardiac troponins are not specific only for cardiac diseases, such as acute myocardial infarction, heart failure, hypertrophic cardiomyopathy, myocarditis, arrhythmias, or aortic valve disease, we can observe elevated values also in non-cardiac conditions, such as chronic kidney disease, anemia, hypertension, amyloidosis, cardiotoxic chemotherapy, pulmonary embolism, sepsis, trauma, drugs (cocaine, amphetamines), stroke, subarachnoid hemorrhage, rhabdomyolysis, or strenuous exercise [23]. In recent years, techniques for the determination of high-sensitivity cardiac troponin (hs-cTn) have been developed, namely, the plasma concentrations of cardiac troponins were significantly lower in patients with heart failure than in patients with acute coronary syndrome [24]. Compared to standard methods, determination of hs-cTn allows more accurate analysis, up to a ten times higher sensitivity of the test, and up to a hundred times lower troponin concentration detection [25]. Measurements of highly sensitive troponins provide additional prognostic information in patients with heart failure, a multimarker approach is recommended, which means the simultaneous determination of a panel of biomarkers, especially in patients with chronic heart failure [26]. In pediatrics, plasma high sensitivity cardiac troponin T (hs-cTnT) may be a useful marker of myocardial damage during chemotherapy in patients with leukemias. Increased levels of hs-cTnT during anthracycline-based therapy are associated with changes in left ventricular myocardial strain [27].

### 3.2. Heart-Type Fatty Acid-Binding Proteins (H-FABPs)

Heart-type fatty acid-binding proteins (H-FABPs) are small intracellular proteins that bind lipids. They are located in tissues with intensive fatty acid metabolism, such as the heart, liver, and intestines [28]. After myocardial damage, heart-type fatty acid-binding proteins (H-FABPs) are released into the blood after twenty minutes, reach a peak in three to four hours, and return to normal within thirty hours [29]. Elevated H-FABP combined with brain natriuretic peptide (BNP) was found the best predictor of mortality and cardiovascular events in patients with heart failure [1,30]. In children with chronic heart failure, elevated H-FABP levels were associated with a poorer prognosis [31].

### 3.3. Glutathione Transferase P1 (GSTP1)

Glutathione transferase P1 (GSTP1) is the most widespread isoenzyme among glutathione transferases and plays a significant role in antioxidant defense [32], it may also act as an inhibitor of tumor necrosis factor-alpha (TNFα) [33]. In patients with heart failure, increased GSTP1 expression is associated with a cellular response to oxidative stress and inflammation [34]. GSTP1 is a more specific predictor of left ventricular function in patients with heart failure compared to N-terminal pro-B-type natriuretic peptide (NT-proBNP). There have been studies on the use of GSTP1 for therapeutic purposes to prevent cardiomyocyte apoptosis after myocardial damage [35]. In pediatrics, GSTP1 is associated with cardiotoxicity in patients treated with doxorubicin. Reduced activity of GSTP1 increases the risk of damaging cardiomyocytes due to reactive oxidative species (ROS) [36].

## 4. Biomarkers of Myocardial Remodeling

Remodeling of the matrix leading to cardiac fibrosis is a crucial factor in the progression of heart failure, as evidenced by impaired systolic and diastolic ventricular function [37].

### 4.1. Galectin-3

Galectin-3 belongs to the group of lectins and allows specific binding of β-galactosides. Galectin-3 is involved in cell adhesion, activation, proliferation, apoptosis, and also in cell migration [38]. In addition, it plays a key role in ventricular remodeling and in the development of fibrosis. In acute decompensated heart failure galectin-3 has a prognostic value for mortality on short-term follow-up [39]. Along with brain natriuretic peptide (BNP), galectin-3 has been shown as a good prognostic factor in heart failure with both preserved and reduced left ventricular ejection fraction [40]. In pediatric patients, galactin-3 can be a predictive factor for the diagnosis and staging of heart failure in children with preserved or reduced ejection fraction [41].

### 4.2. Soluble Isoform of Suppression of Tumorigenicity 2 (sST2)

**Figure 2:**
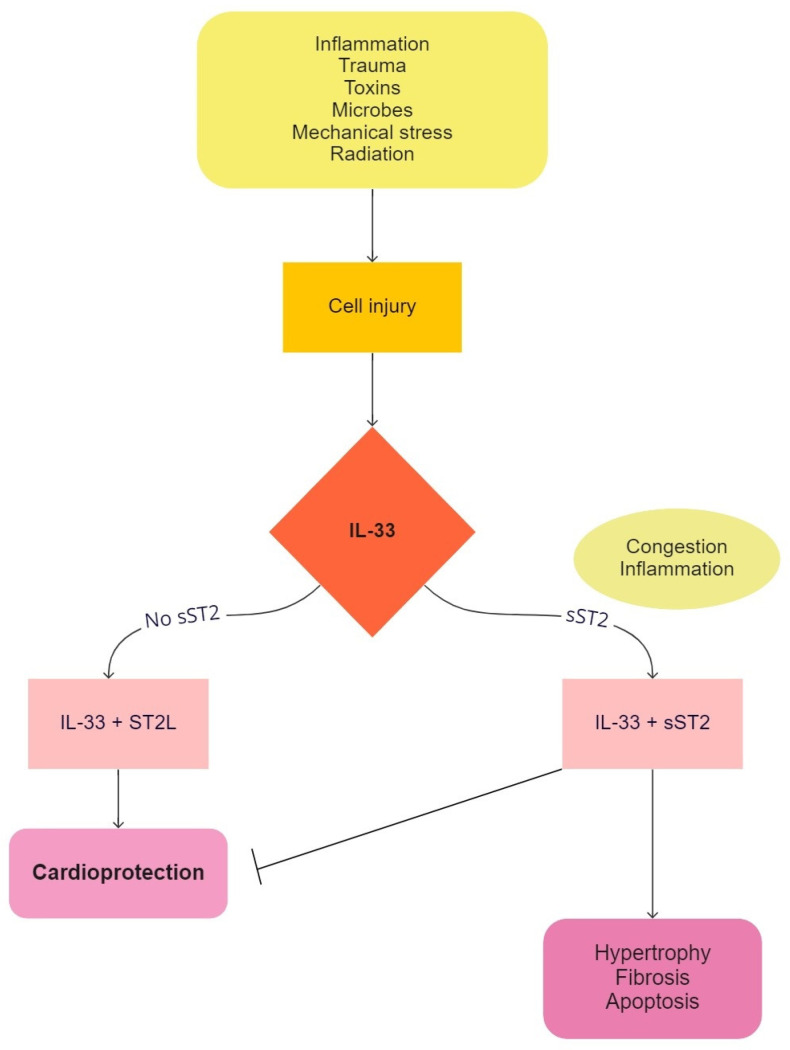
The interaction between interleukin-33 (IL-33) and the transmembrane isoform of suppression of tumorigenicity 2 ligand (ST2L) in response to myocardial stress, which leads to the cardioprotective function of suppression of tumorigenicity 2 (ST2). The binding of a soluble isoform of suppression of tumorigenicity 2 (sST2) to IL-33 inhibits the interaction between IL-33 and ST2L and reduces the effects of IL-33 on the inflammatory system, which minimizes the cardioprotective function of ST2.

The soluble isoform of tumor suppression 2 or sST2 has become an interesting biomarker of heart failure due to its involvement in the processes of inflammation, fibrosis, and strain on the heart muscle. There are two isoforms of ST2 protein, namely the transmembrane isoform ST2L and the soluble isoform sST2. The transmembrane isoform has an immunomodulatory function via the interleukin-33 (IL-33) signaling pathway [42]. Mechanical stretching of cardiac fibroblasts and cardiomyocytes activates the signaling pathway via IL-33, which prevents hypertrophy of the cardiomyocytes. The interaction between IL-33 and ST2 is up-regulated as a response to myocardial stress and has a cardioprotective role [43]. On the other side, the soluble isoform of ST2 protein (sST2) reduces the cardioprotective effect of IL-33 by acting as a decoy receptor, as seen in Figure 2 [2]. Elevated sST2 levels are found in inflammatory and malignant diseases, as well as in heart failure as a result of congestion and inflammation [44]. Serial determination of sST2 plays a significant predictive role in ventricular remodeling, and in the deterioration of heart failure [45], it also has a role over traditional biomarkers for determining the prognosis of heart failure [16]. Compared to natriuretic peptides, sST2 does not depend on age, body mass index, or renal function [46].

### 4.3. MicroRNAs

MicroRNAs are small non-coding RNAs that are part of myocardial remodeling processes resulting in cardiac hypertrophy and fibrosis, therefore, they are involved in the development and progression of heart failure [47]. Consequently, they are becoming increasingly valuable as a potential biomarker for guiding the therapy of heart failure [48]. Moreover, studies have shown that combinations of microRNAs can be used to differentiate heart failure with preserved ejection fraction from heart failure with reduced ejection fraction [49]. However, there are still some limitations to their clinical use, especially due to variable measurements and unclear pathophysiological role [50]. In pediatrics, microRNA is a predictive factor for long-term outcomes in children with dilated cardiomyopathy [51].

## 5. Biomarkers of Inflammation

Chronic inflammation is one of the pivotal mechanisms in developing heart failure and is related to the progression and prognosis of heart failure. Inflammatory mediators have a direct impact on the heart muscle as well as on the adrenergic system, which leads to hypertrophy, fibrosis, and impaired cardiac function [1]. In the group of inflammatory biomarkers of heart failure are some traditional biomarkers, such as C-reactive protein (CRP) and high-sensitivity C-reactive protein (hs-CRP), tumor necrosis factor-alpha (TNF-α), interleukin-6 (IL-6), as well as some new biomarkers [5]. Anti-inflammatory therapies are under investigation in patients with heart failure [52].

### 5.1. Growth Differentiation Factor-15 (GDF-15)

Growth differentiation factor-15 (GDF-15) is a multifunctional cytokine that is a part of transforming growth factors β and also has anti-hypertrophic effects [47]. Increased expression of GDF-15 has a cardioprotective function, which has been observed in heart failure, atherosclerosis, and endothelial dysfunction. GDF-15 has been associated with inflammation, malignancies, lung disease, diabetes mellitus, and kidney disease [53]. GDF-15 is promising as a prognostic factor in patients with heart failure with preserved ejection fraction [54]. Research on the therapeutic options of GDF-15 is also ongoing, regarding the clinical usefulness of targeting GDF-15 in cardiometabolic diseases [55]. In pediatrics, the level of GDF-15 is positively related to the degree of cardiac function in patients with congenital heart disease (CHD) [56].

### 5.2. Endothelial Microparticles (EMPs) and Endothelial Progenitor Cells (EPCs)

Endothelial microparticles (EMPs) and endothelial progenitor cells (EPCs) are related to impaired endothelial function and systemic inflammation [47]. Clinical studies have shown a connection between the EMP and EPC ratio and the stage of heart failure. However, it is not clear if it is a good predictor for guiding treatment [57].

## 6. Biomarkers of Renal Dysfunction

Cardiovascular and renal diseases are strongly related, as impaired function of one organ often leads to deterioration of function of the other. In patients with cardiorenal syndrome, i.e., with the involvement of both organ systems, morbidity and mortality are greatly increased. Many biomarkers are already used in the field of renal impairment, such as cystatin C, NGAL (neutrophil gelatinase-associated lipocalin), KIM-1 (kidney injury molecule-1), interleukin-18, L-FABP (liver-type fatty acid-binding protein), NAG (N-acetyl-β-D-glucosaminidase), β-2 microglobulin, and glutathione-S-transferase [58]. In the case of heart failure, panels of renal dysfunction biomarkers are often used [47].

### 6.1. Neutrophil Gelatinase-Associated Lipocalin (NGAL)

Neutrophil gelatinase-associated lipocalin (NGAL) is also known as lipocalin-2 and is one of the lipocalins that are small extracellular proteins associated with inflammation, the transport of small hydrophobic ligands such as steroids and lipids, and prostaglandin synthesis [59]. NGAL also acts as a growth and differentiation factor in the renal epithelium [60]. Moreover, it is used to detect early renal dysfunction and it is also considered to be an independent predictor of heart failure [61]. Urinary NGAL concentrations are elevated in patients with heart failure and impaired renal function, furthermore, NGAL is a good independent prognostic factor in patients with heart failure [62], or as an added prognostic factor along with brain natriuretic peptide (BNP), as shown in the GALLANT trial [16,63]. Data about plasma levels of NGAL in children are limited. In heart failure caused by dilated cardiomyopathy, NGAL levels were significantly increased, however, there was no significant relationship between plasma levels of NGAL and myocardial function or clinical presentation [64].

### 6.2. Kidney Injury Molecule-1 (KIM-1)

Kidney injury molecule-1 (KIM-1) is a transmembrane glycoprotein found on the apical membrane of proximal tubules in patients with renal impairment, on the other hand, in the case of healthy kidneys, KIM-1 gene expression is not found [65]. KIM-1 affects the repair of damaged kidney tissue [66]. KIM-1 is a receptor on renal epithelial cells responsible for conversion of normal tubule cells into phagocytes. In addition to its important role as a biomarker of acute renal impairment, KIM-1 is also used as a biomarker of renal tubular impairment in patients with acute and chronic heart failure [67]. Furthermore, KIM-1 correlates well with the stage of heart failure and can be used as a predictor of the cardiorenal syndrome [68]. Urine KIM-1 may be a good biomarker for the early prediction of acute kidney injury after open cardiac surgery in children with congenital heart disease (CHD) [69].

## 7. Biomarkers of Neurohumoral Activation

Cardiac failure is characterized by the activation of the neurohumoral system, namely the sympathetic nervous system. At the initial stage of heart failure, the body tries to provide adequate tissue perfusion through compensatory mechanisms, including activation of the sympathetic nervous system, renin-angiotensin-aldosterone system, decreased activity of the parasympathetic system, and dysregulation of the signaling pathway with nitric oxide (NO), and synthesis of inflammatory cytokines [70]. In addition to the classic biomarkers of neurohumoral activation, such as norepinephrine, plasma renin activity, angiotensin II and aldosterone, newer biomarkers are also the subject of research.

### 7.1. Adrenomedullin (MR-proADM)

Adrenomedullin (ADM) is a peptide found in the highest concentrations in the adrenal medulla, ventricles, kidneys, and lungs [71]. Endothelial cells actively synthesize and secrete ADM with a vasodilatory effect. Plasma concentration of ADM correlates well with the stage of heart failure, left ventricular ejection fraction, left ventricular diastolic dysfunction, and pulmonary artery pressure [72]. Due to the short half-life of ADM and its instability in plasma, a more stable form, namely mid-regional pro-adrenomedullin (MR-proADM) is used in laboratory analysis. Elevated levels of MR-proADM are strongly associated with the presence of chronic heart failure [16]. In addition, MR-proADM is a highly sensitive predictor of mortality in patients with heart failure, but on the other hand, poorly specific due to its wide tissue distribution and elevated values in many other diseases [73]. ADM is also an independent prognostic factor of heart failure in children [74].

### 7.2. Copeptin

Copeptin is a quantitative biomarker of endogenous biomechanical stress and plays a significant role in body water homeostasis through renal reabsorption, blood volume regulation, osmolality, and vasoconstriction. It is also important in myocardial contractility, cell proliferation, and antidiuretic hormone activity [70]. Although plasma levels are very variable, copeptin is usually elevated in severe hypertension, acute and chronic heart failure, myocardial infarction, stroke, diabetes mellitus, and advanced kidney diseases [47]. In some studies, copeptin was even superior to brain natriuretic peptide (BNP) or N-terminal-proBNP (NT-proBNP) as a predictor of mortality and staging of heart failure, but on the other hand, the markers seem to be closely related with similar predictive properties in several studies. Furthermore, copeptin is associated with higher laboratory costs and is not available in all institutions [75]. In pediatrics, the copeptin level is elevated in children with heart failure due to cardiomyopathies [76].

### 7.3. Matrix Metalloproteinases (MMPs)

Matrix metalloproteinases (MMPs) are part of neurohormonal modulation and act also as an activator of the inflammatory system. They have a significant role in the accumulation of extracellular collagen and in the development of fibrosis. The imbalance between expression of MMPs and suppression of their tissue inhibitors may lead to impaired cardiac function and progression of heart failure [47]. Metalloproteinase-9 (MMP-9) may be an independent marker for suspecting and predicting the development of heart failure in children with rheumatic heart disease, furthermore, the level of MMP-9 correlates well with the severity of heart failure [77].

## 8. Biomarkers of Oxidative Stress

Heart failure relates to oxidative stress due to circulating neurohormones, hemodynamic changes, inflammation, and poor oxygen supply. Then, disorders of redox balance even further impair vital structures and affect signaling pathways of cell renewal and cell death, additionally deteriorating heart failure [78]. There are many biomarkers in the group of oxidative stress, such as serum uric acid, myeloperoxidase (MPO), vitamin D3, ceruloplasmin, and 8-hydroxy-2-0-deoxyguanosine. An elevated level of serum uric acid is common in patients with heart failure, hypertension, atherosclerosis, obesity, diabetes mellitus, and chronic renal disease [79]. Serum levels of myeloperoxidase, vitamin D3, ceruloplasmin, and 8-hydroxy-2-0-deoxyguanosine correlate well with the stage of heart failure [47].

### 8.1. Ceruloplasmin

Ceruloplasmin is a plasma glycoprotein synthesized in the liver. It is considered as an acute phase protein, and it is also involved in copper transport. Ceruloplasmin has a pro-oxidative and antioxidative role [80]. Elevated levels of ceruloplasmin are associated with the general inflammation status of the body and help identify individuals at higher risk of developing heart failure or disease worsening [81].

### 8.2. Myeloperoxidase (MPO)

Myeloperoxidase (MPO) is one of the enzymes found in neutrophils that allows the synthesis of chloric acid and free radicals involved in the destruction of phagocytic pathogens [82]. MPO is used as an independent predictor of heart failure and cardiovascular events [83]. In addition, it can be used in combination with other biomarkers, such as brain natriuretic peptide (BNP) and C-reactive protein (CRP). As part of a larger prospective study on randomly selected individuals, MPO, CRP, and BNP in plasma and urine were determined. The combination of these three biomarkers was a good predictor of left ventricular systolic dysfunction [84]. Obesity is associated with chronic low-grade inflammation and cardiovascular risk. The elevated level of MPO in the serum of prepubertal and pubertal obese individuals is considered to be a predictor of cardiovascular complications [85].

## 9. Biomarkers Determined in Urine

With the development of extended research on biomarkers in patients with heart failure and cardiovascular risk, the possibility to determine biomarkers in urine has emerged, which further simplifies investigation and improves the compliance of patients and healthy individuals in clinical trials, even more so in pediatrics. Biomarkers of renal dysfunction associated with heart failure, such as neutrophil gelatinase-associated lipocalin (NGAL) and kidney injury molecule-1 (KIM-1), may be determined in urine. NGAL is a good independent prognostic factor in patients with heart failure [62]. KIM-1 is used as a biomarker of renal tubular impairment in patients with acute or chronic heart failure, furthermore, it correlates well with the stage of disease and may also be used as a predictor of cardiorenal syndrome [68]. Galectin-3, which is involved in myocardial remodeling and fibrosis, can also be detected in urine and has been shown as a good prognostic factor in patients with heart failure with preserved ejection fraction [86]. In addition, natriuretic peptides, which are the main biomarkers of myocardial stretch may also be determined in the urine, as well as sodium concentration, β-2 microglobulin, and albumin/creatinine ratio in order to achieve risk stratification of patients with heart failure [87].

## 10. Future Perspectives

With the development of medicine and bioinformatics, attempts have been made to determine which combination of biomarkers has the best predictive value in the diagnosis, prognosis, and monitoring of patients with heart failure. The future use of biomarkers is reflected in multimarker panels, which include biomarkers with different pathophysiological mechanisms that contribute to the development of heart failure, thereby increasing their diagnostic and prognostic predictive value [1]. Responses to the pharmacotherapies vary widely in patients with heart failure, therefore, up-titration of medicines based on biomarkers is becoming increasingly useful for successful treatment. The subject of ongoing research are angiotensin-converting enzyme inhibitors, angiotensin receptor blockers, beta-blockers, and mineralocorticoid receptor antagonists [88]. Recently, there have been shifts in the treatment of heart failure with sodium-glucose cotransporter 2 (SGLT2) inhibitors, which are antihyperglycemic agents primarily used as an antidiabetic therapy. SGLT2 inhibitors improve cardiovascular and renal outcomes, although the cardioprotective effects of SGLT2 inhibitors remain incompletely understood in patients with heart failure. The favorable effects of SGLT2 inhibitors occur independent of blood glucose lowering [89]. Pharmacogenetics is the next step to individualized medicine through identifying genetic variants in patients with heart failure, who consequently are most likely to benefit from certain pharmacotherapy [90]. In pediatrics, cardiomyopathies represent an important cause of heart failure. Genetic testing for cardiomyopathies has been already established and provides diagnosis, prognosis, risk stratification, as well as early identification and initiation of therapies [91].

## 11. Conclusions

The number of potential biomarkers of heart failure is increasing over recent years and is a current subject of ongoing research. Biomarkers reflect different pathophysiological mechanisms that are present in heart failure. Due to the high prevalence of heart failure and many systemic diseases that can indirectly affect the heart muscle, research in the field of biomarkers has been underway for many years to identify as accurately as possible the risk of disease development and progression, cardiovascular events, and the associated need for close monitoring of risk groups. Newer biomarkers have certain prognostic advantages, but also some limitations, especially in pediatrics due to poor specificity and insufficient data on long-term monitoring. Therefore, the predictive value of these biomarkers is not fully confirmed and requires further investigation for widespread clinical use. Multimarker panels are becoming increasingly promising, however, more clinical trials are required to improve the understanding of individualized therapy of heart failure under biomarker control. Hopefully, the discoveries of novel biomarkers and their treatment target may lead to improved care for patients with heart failure.

## Figures and Tables

**Table 1:** Biomarkers of heart failure based on pathophysiological mechanisms.

Myocardial Stretch	Myocyte Injury	Myocardial Remodeling	Inflammation	Renal Dysfunction	Neurohumoral Activation	Oxidative Stress
BNP ^1^	CTn ^5^ (TnI ^6^, TnT ^7^)	galectin-3	GDF-15 ^12^	NGAL ^19^	MR-proADM ^24^	ceruloplasmin
NT-proBNP ^2^	hs-cTn ^8^	sST2 ^11^	EMPs ^13^	KIM-1 ^20^	copeptin	MPO ^26^
ANP ^3^	H-FABPs ^9^	microRNAs	EPCs ^14^	cystatin C	MMPs ^25^	SUA ^27^
MR-proANP ^4^	GSTP1 ^10^		CRP ^15^	IL-18 ^21^		vitamin D3
			hs-CRP ^16^	L-FABP ^22^		8-hydroxy-2-0-deoxyguanosine
			TNF-α ^17^	NAG ^23^		
			IL-6 ^18^	β-2 microglobulin		
				glutathione-S-transferase		

^1^ BNP, brain natriuretic peptide; ^2^ NT-proBNP, N-terminal-proBNP; ^3^ ANP, atrial natriuretic peptide; ^4^ MR-proANP, mid-regional proatrial natriuretic peptide; ^5^ cTn, cardiac troponins; ^6^ TnI, troponin I; ^7^ TnT, troponin T; ^8^ hs-cTn, high-sensitivity cardiac troponin; ^9^ H-FABPs, heart-type fatty acid-binding proteins; ^10^ GSTP1, glutathione transferase P1; ^11^ sST2, soluble isoform of suppression of tumorigenicity 2; ^12^ GDF-15, growth differentiation factor-15; ^13^ EMPs, endothelial microparticles; ^14^ EPCs, endothelial progenitor cells; ^15^ CRP, C-reactive protein; ^16^ hs-CRP, high-sensitivity C-reactive protein; ^17^ TNF-α, tumor necrosis factor alpha; ^18^ IL-6, interleukin-6; ^19^ NGAL, neutrophil gelatinase-associated lipocalin; ^20^ KIM-1, kidney injury molecule-1; ^21^ IL-18, interleukin-18; ^22^ L-FABP, liver-type fatty acid-binding protein; ^23^ NAG, N-acetyl-β-D-glucosaminidase; ^24^ MR-proADM, mid-regional pro-adrenomedullin; ^25^ MMPs, matrix metalloproteinases; ^26^ MPO, myeloperoxidase; ^27^ SUA, serum uric acid.

**Table 2:** Normal values of some biomarkers of heart failure with pediatric specificities according to available data.

Biomarker	Adult Population	Pediatric Population
BNP ^1^	<35 ng/L	
NT-proBNP ^2^	<125 ng/L	<3569 ng/L (0–1 Y ^11^) <178 ng/L (1–19 Y)
MR-proANP ^3^	<40 pmol/L	
HsTnT ^4^	<14 ng/L	<78 ng/L (0–6 M ^12^) <34 ng/L (6 M–1 Y) <6 ng/L (1–19 Y)
HsTnI ^5^	<6 ng/L	<93.8 ng/L (<1 M) <52.1 ng/L (1–12 M) <48.1 ng/L (1–12 Y) <3.9 ng/L (13–18 Y)
H-FABPs ^6^	<19 ng/mL	
Galectin-3	<22.1 ng/mL	<33 ng/mL
sST2 ^7^	<49.3 ng/mL (male) <33.5 ng/mL (female)	<50 ng/mL
GDF-15 ^8^	<584 pg/mL	
NGAL ^9^	<50 ng/mL	
MR-proADM ^10^	<0.55 nmol/L	
Copeptin	<11.25 pmol/L	<13.1 pmol/L

^1^ BNP, brain natriuretic peptide; ^2^ NT-proBNP, N-terminal-proBNP; ^3^ MR-proANP, mid-regional proatrial natriuretic peptide; ^4^ hsTnT, high-sensitivity troponin T; ^5^ hsTnI, high-sensitivity troponin I; ^6^ H-FABPs, heart-type fatty acid-binding proteins; ^7^ sST2, soluble isoform of suppression of tumorigenicity 2; ^8^ GDF-15, growth differentiation factor-15; ^9^ NGAL, neutrophil gelatinase-associated lipocalin; ^10^ MR-proADM, mid-regional pro-adrenomedullin; ^11^ Y, year; ^12^ M, month.

## Data Availability

All the data are available within the article.
